# *TRIB1* and *TRPS1* variants, G × G and G × E interactions on serum lipid levels, the risk of coronary heart disease and ischemic stroke

**DOI:** 10.1038/s41598-019-38765-7

**Published:** 2019-02-20

**Authors:** Qing-Hui Zhang, Rui-Xing Yin, Wu-Xian Chen, Xiao-Li Cao, Jin-Zhen Wu

**Affiliations:** 1 Department of Cardiology, Institute of Cardiovascular Diseases, The First Affiliated Hospital, Guangxi Medical University, Nanning, 530021 Guangxi People’s Republic of China; 2 Department of Neurology, The First Affiliated Hospital, Guangxi Medical University, Nanning, 530021 Guangxi People’s Republic of China

## Abstract

This study aimed to assess the association of the tribbles pseudokinase 1 (*TRIB1*) and transcriptional repressor GATA binding 1 (*TRPS1*) single nucleotide polymorphisms (SNPs) and the gene-gene (G × G) and gene-environment (G × E) interactions with serum lipid levels, the risk of coronary heart disease (CHD) and ischemic stroke (IS) in the Guangxi Han population. Genotyping of the rs2954029, rs2980880, rs10808546, rs231150, rs2737229 and rs10505248 SNPs was performed in 625 controls and 1146 unrelated patients (CHD, 593 and IS, 553). The genotypic and allelic frequencies of some SNPs were different between controls and patients (CHD, rs2954029 and rs231150; IS, rs2954029 and rs2980880; *P* < 0.05-0.01). Two SNPs were associated with increased risk of CHD (rs2954029 and rs231150) and IS (rs2954029) in different genetic models. Several SNPs in controls were associated with total cholesterol (rs2954029, rs2980880 and rs2737229), triglyceride (rs2954029 and rs10808546), low-density lipoprotein cholesterol (rs2954029), high-density lipoprotein cholesterol (rs2980880 and rs231150) and apolipoprotein A1 (rs2737229) levels. The rs2954029TA/AA-age (>60 year) interaction increased the risk of CHD, whereas the rs10808546CT/TT-drinking interaction decreased the risk of IS. The rs2954029A-rs2980880C-rs10808546C haplotype was associated with increased risk of CHD and IS. The rs2954029A-rs2980880T-rs10808546C haplotype was associated with increased risk of CHD. The rs2954029-rs231150 interactions had an increased risk of both CHD and IS. These results suggest that several *TRIB1* and *TRPS1* SNPs were associated with dyslipidemia and increased risk of CHD and IS in our study population. The G × G and G × E interactions on serum lipid levels, and the risk of CHD and IS were also observed.

## Introduction

Coronary heart disease (CHD) and ischemic stroke (IS) remain the leading causes of morbidity and mortality worldwide^1,2^, resulting in a substantial economic and social burden^3^. The main pathological basis of both diseases is atherosclerosis^4^, which is characterized by the accumulation of lipid and inflammatory immune processes in the arterial wall^5^. Therefore, CHD and IS may share some common genetic and environmental determinants such as sex, age, dyslipidemia, hypertension, drinking, smoking and body mass index (BMI)^6,7^. Many genes and loci in previous genome-wide association studies (GWASes) have been shown to be predisposed to CHD^8^ or IS^9^ in different populations. Furthermore, some genetic mutations originally identified to be associated with CHD were also subsequently showed to influence the risk of IS^10–12^. Family history and twin studies showed that almost 30–60% of the incidence of CHD and IS^13^ can be explained by genetic factors, suggesting a considerable genetic contribution.

The tribbles pseudokinase 1 (*TRIB1*) and transcriptional repressor GATA binding 1 (*TRPS1*) genes are closely located on chromosome 8q. The *TRIB1* encodes the tribbles homolog 1 protein, which is recognized as modulator of many fundamental signalling pathways, biological processes and disease pathologies^14,15^. The involvement of *TRIB1* in hepatic lipid metabolism has been validated through viral-mediated hepatic overexpression of the gene in mice; increasing levels of *TRIB1* decreased plasma lipids in a dose-dependent manner. The *TRPS1* encodes a transcription factor interacted with a dynein light chain protein, which lower the binding to GATA consensus sequences, thereby suppressing its transcription activity^16,17^. Many GWASes and target single nucleotide polymorphism (tag SNP) studies have showed that several common SNPs in *TRIB1*^18–22^ and *TRPS1*^18,19,23,24^ have been significantly associated with multiple plasma lipid traits and cardiovascular disease in different populations. However, little is known about such association in the Chinese populations. Therefore, using the method of tag SNPs combined with recent research reports, we detected six SNPs in the *TRIB1* (rs2954029, rs2980880 and rs10808854) and *TRPS1* (rs231150, rs2737229 and rs10505248), their G × G and G × E interactions on serum lipid levels, the risk of CHD and IS in a Southern Chinese Han population.

## Results

### General characteristics of the subjects

The general characteristics of the patients and healthy controls are summarized in Table 1. The values of BMI, systolic blood pressure, pulse pressure, and triglyceride (TG) were higher but serum total cholesterol (TC), high-density lipoprotein cholesterol (HDL-C), apolipoprotein (Apo) A1, the percentages of subjects who consumed alcohol, and the ratio of ApoA1 to ApoB were lower in CHD patients than in controls (*P* < 0.001 for all). The values of BMI, systolic blood pressure, diastolic blood pressure, pulse pressure, and TG were higher but TC, HDL-C, ApoA1, the percentages of subjects who consumed alcohol, and the ratio of ApoA1 to ApoB were lower in IS patients than in controls (*P* < 0.001 for all).

**Table 1:** Comparison of general characteristics and serum lipid levels between controls and patients. CHD, coronary heart disease; IS, ischemic stroke; HDL-C, high-density lipoprotein cholesterol; LDL-C, low-density lipoprotein cholesterol.

Parameter	Control	CHD	IS	*P*_CHD_	*P*_IS_
Number	625	593	553	—	—
Male/female	462/163	425/168	408/145	0.207	0.504
Age, years	61.85 ± 11.87	62.81 ± 12.28	62.21 ± 10.54	0.162	0.578
Body mass index, kg/m^2^	22.62 ± 2.82	23.39 ± 3.46	23.95 ± 3.21	0.000	0.000
Systolic blood pressure, mmHg	128.43 ± 19.00	133.03 ± 23.46	147.56 ± 21.87	0.000	0.000
Diastolic blood pressure, mmHg	80.63 ± 11.39	79.27 ± 14.16	83.65 ± 12.84	0.064	0.000
Pulse pressure, mmHg	47.80 ± 13.75	53.76 ± 17.58	63.91 ± 17.68	0.000	0.000
Cigarette smoking, n (%)	244(39.0)	252(42.5)	230(41.6)	0.121	0.203
Alcohol consumption, n (%)	265 (42.4)	153(25.8)	170(30.7)	0.000	0.000
Total cholesterol, mmol/L	4.89 ± 1.04	4.54 ± 1.21	4.54 ± 1.16	0.000	0.000
Triglyceride, mmol/L	0.99 (0.65)	1.35(0.95)	1.36(0.94)	0.000	0.000
HDL-C, mmol/L	1.90 ± 0.48	1.14 ± 0.34	1.23 ± 0.41	0.000	0.000
LDL-C, mmol/L	2.73 ± 0.76	2.72 ± 1.02	2.68 ± 0.91	0.836	0.320
Apolipoprotein (Apo) A1, g/L	1.41 ± 0.26	1.04 ± 0.53	1.03 ± 0.22	0.000	0.000
ApoB, g/L	0.90 ± 0.21	0.91 ± 0.27	0.89 ± 0.24	0.659	0.610
ApoA1/ApoB	1.65 ± 0.56	1.37 ± 2.43	1.26 ± 0.59	0.005	0.000

The value of triglyceride was presented as median (interquartile range), the difference between CHD/IS patients and controls was determined by the Wilcoxon-Mann-Whitney test. *P*_CHD_, CHD *vs*. controls; *P*_IS_, IS *vs*. controls.

### Genotypic and allelic frequencies in controls and patients

**Figure 1:**
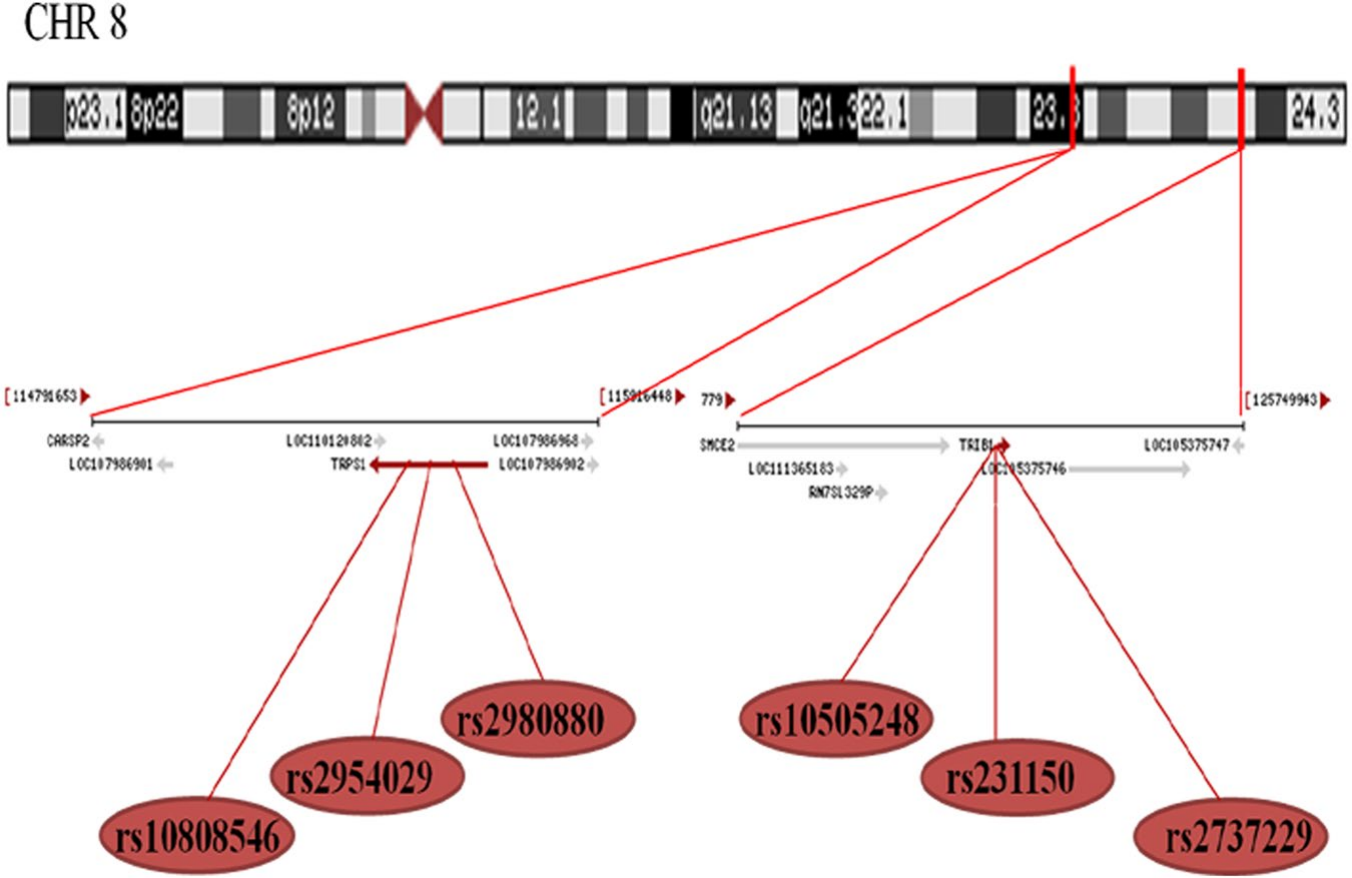
The positions of the *TRIB1* and *TRPS1* SNPs. *TRIB1*, tribbles pseudokinase 1; *TRPS1*, transcriptional repressor GATA binding 1.

**Figure 2:**
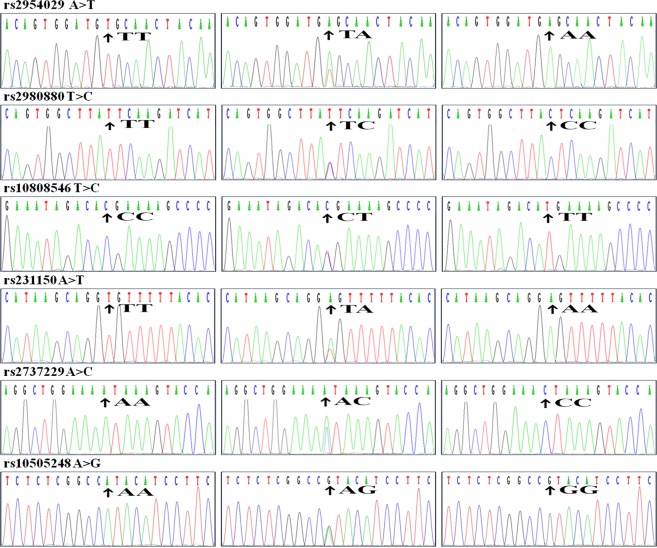
The parts of the nucleotide direct sequencing results of the *TRIB1* and *TRPS1* SNPs. *TRIB1*, tribbles pseudokinase 1; *TRPS1*, transcriptional repressor GATA binding 1.

Six SNPs in this motif are closely located on chromosome 8q (Fig. 1) and the genotypes were detected by polymerase chain reaction and restriction fragment length polymorphism (PCR-RFLP) were also confirmed by direct sequencing (Fig. 2). The genotypic and allelic frequencies of the 6 SNPs in *TRIB1* and *TRPS1* are presented in Table 2. The genotype distribution of the 6 SNPs was concordant with the Hardy-Weinberg equilibrium (HWE) in patients and controls (*P* > 0.05 for all). The genotypic and allelic frequencies of the rs2954029 and rs231150 were different between controls and CHD patients (*P* < 0.05 for all). The genotypic and allelic frequencies of the rs2954029 and rs2980880 SNPs were different between controls and IS patients (*P* < 0.05 for all). The frequencies of rs2954029A allele and rs2954029AA genotype were higher in CHD (A, 43.7%; AA, 16.9%) or IS (A, 44.8%; AA, 18.6%) patients than in control subjects (A, 38.5.6%; AA, 13.4%; *P* < 0.05 for all). The frequencies of rs231150A allele and rs231150AA genotype were higher in CHD (A, 47.4%; AA, 20.7%) than in control subjects (A, 41.1.6%; AA, 16.0%; *P* < 0.01).

**Table 2:** Genotypic and allelic frequencies of six SNPs in controls and patients (n (%)) SNP, single nucleotide polymorphism; CHD, coronary heart disease; IS, ischemic stroke; HWE, Hardy-Weinberg equilibrium.

SNP/Genotype	Control	CHD	IS	Allele	Control	CHD	IS
	n = 625	n = 593	n = 553				
**rs2954029**							
TT	228 (36.5)	175 (29.5)	160 (28.9)				
TA	313 (50.1)	318 (53.6)	290 (52.4)	T	769 (61.5)	668 (56.3)	610 (55.2)
AA	84 (13.4)	100 (16.9)	103 (18.6)	A	482 (38.5)	518 (43.7)	496 (44.8)
*x* ^2^		7.566	10.363	*x* ^2^		6.67	9.798
*P*		0.023	0.006	*P*		0.010	0.002
*P*_HWE_	0.149	0.104	0.158				
**rs2980880**							
TT	339 (54.2)	311 (52.4)	266 (48.1)				
TC	238 (38.1)	230 (38.8)	226 (40.9)	T	916 (73.2)	852 (71.8)	758 (68.5)
CC	48 (7.7)	52 (8.8)	61 (11.0)	C	334 (26.8)	334 (28.2)	348 (31.5)
*x* ^2^		0.663	6.292	*x* ^2^		0.636	6.423
*P*		0.718	0.043	*P*		0.425	0.011
*P*_HWE_	0.490	0.313	0.218				
**rs10808546**							
CC	437 (69.9)	413 (69.6)	400 (72.3)				
CT	164 (26.2)	160 (27.0)	135 (24.4)	T	1038 (73.2)	986 (83.1)	935 (84.5)
TT	24 (3.8)	20 (3.4)	18 (3.3)	C	212 (17.0)	200 (16.9)	171 (26.0)
*x* ^2^		0.250	0.908	*x* ^2^		0.004	0.968
*P*		0.882	0.635	*P*		0.949	0.325
*P*_HWE_	0.087	0.358	0.120				
**rs231150**							
TT	211 (33.8)	154 (26.0)	161 (29.1)				
TA	314 (50.2)	316 (53.3)	293 (53.0)	A	736 (58.9)	624 (52.6)	615 (55.6)
AA	100 (16.0)	123 (20.7)	99 (17.9)	T	514 (41.1)	562 (47.4)	491 (44.4)
*x* ^2^		10.446	3.063	*x* ^2^		9.690	2.572
*P*		0.005	0.216	*P*		0.002	0.109
*P*_HWE_	0.348	0.094	0.085				
**rs2737229**							
AA	258 (41.3)	254 (42.8)	241 (43.6)				
AC	300 (48.0)	279 (47.0)	254 (45.9)	A	816 (56.3)	787 (66.4)	736 (66.5)
CC	67 (10.7)	60 (10.1)	58 (10.5)	T	434 (43.7)	399 (33.6)	370 (33.5)
*x* ^2^		0.338	0.648	*x* ^2^		0.314	0.418
*P*		0.844	0.723	*P*		0.575	0.518
*P*_HWE_	0.141	0.191	0.547				
**rs10505248**							
AA	463 (74.1)	427 (72.0)	415 (75.0)				
AG	144 (23.0)	148 (25.0)	125 (22.6)		1051 (84.1)	990 (83.5)	946 (85.5)
GG	18 (2.9)	18 (3.0)	13 (2.4)		199 (15.9)	196 (16.5)	160 (14.5)
*x* ^2^		0.671	0.373	*x* ^2^		0.165	0.960
*P*		0.715	0.830	*P*		0.685	0.327
*P*_HWE_	0.102	0.243	0.331				

### Genotypes and the risk of CHD and IS

As shown in Table 3, the genotypes of the rs2954029 and rs231150 SNPs were associated with increased risk of CHD after the Bonferroni correction (a value of *P* < 0.008 was considered statistically significant) in different genetic model: co-dominant model (rs2954029, AA *vs*. TT, OR = 1.80, 95% CI = 1.34–2.62, *P* = 0.0024); dominant models (rs2954029, TA/AA *vs*. TT, OR = 1.52, 95% CI = 1.18–1.97, *P* = 0.0011; rs231150, TA/AA *vs*. TT, OR = 1.48, 95% CI = 1.14–1.92, *P* = 0.0035) and log-additive model (rs2954029, A *vs*. T, OR = 1.36, 95% CI = 1.14–1.63, *P* < 0.001; rs231150, A *vs*. T, OR = 1.36, 95% CI = 1.14–1.63, *P* < 0.0015).

**Table 3:** Genotypes of the six *TRIB1* and *TRPS1* SNPs and the risk of CHD and IS.

SNP/Model	Ref. Genotype	Effect Genotype	CHD (OR 95% CI)	*P*_CHD_	IS (OR 95% CI)	*P*_IS_
**rs2954029**						
Codominant	TT	TA	1.45 (0.93–1.90)	0.0024*	1.44 (1.10–1.88)	0.0091
		AA	1.80 (1.34–2.62)		1.60 (1.11–2.31)	
Dominant	TT	TA/AA	1.52 (1.18–1.97)	0.0011*	1.48 (1.14–1.90)	0.0026*
Recessive	TT/TA	AA	1.43 (1.02–2.00)	0.035	1.28 (0.92–1.77)	0.14
Overdominant	TT/AA	TA	1.21 (0.95–1.53)	0.12	1.24 (0.98–1.57)	0.078
Log-additive			1.36 (1.14–1.63)	6E-04*	1.30 (1.09–1.55)	0.0039*
**rs2980880**						
Codominant	TT	TC	1.12 (0.87–1.45)	0.23	1.21 (0.94–1.55)	0.13
		CC	1.45 (0.93–2.29)		1.46 (0.95–2.24)	
Dominant	TT	TC/CC	1.17 (0.92–1.49)	0.19	1.25 (0.98–1.59)	0.067
Recessive	TT/TC	CC	1.38 (0.89–2.14)	0.15	1.35 (0.89–2.04)	0.16
Overdominant	TT/CC	TC	1.07 (0.84–1.37)	0.59	1.14 (0.89–1.45)	0.30
Log-additive			1.17 (0.97–1.41)	0.1	1.21 (1.01–1.45)	0.043
**rs10808546**						
Codominant	CC	CT	0.97 (0.74–1.27)	0.89	0.88 (0.67–1.16)	0.58
		TT	0.87 (0.45–1.66)		0.82 (0.43–1.57)	
Dominant	CC	CT/ TT	0.95 (0.74–1.24)	0.72	0.87 (0.67–1.13)	0.31
Recessive	CC /CT	TT	0.87 (0.46–1.67)	0.68	0.85 (0.45–1.62)	0.62
Overdominant	CC/ TT	CT	0.97 (0.74–1.27)	0.84	0.89 (0.68–1.17)	0.39
Log-additive			0.95 (0.76–1.19)	0.66	0.89 (0.71–1.11)	0.30
**rs231150**						
Codominant	TT	TA	1.40 (1.06–1.84)	0.018	1.16 (0.89–1.52)	0.50
		AA	1.74 (1.12–2.49)		1.18 (0.82–1.70)	
Dominant	TT	TA/AA	1.48 (1.14–1.92)	0.0035*	1.17 (0.90–1.51)	0.24
Recessive	TT/TA	AA	1.40 (1.03–1.91)	0.032	1.07 (0.78–1.48)	0.66
Overdominant	TT/AA	TA	1.13 (0.89–1.43)	0.31	1.10 (0.86–1.39)	0.45
Log-additive			1.33 (1.11–1.59)	0.0015*	1.10 (0.92–1.31)	0.29
**rs2737229**						
Codominant	AA	AC	0.90 (0.70–1.16)	0.68	0.90 (0.70–1.16)	0.62
		CC	0.88 (0.58–1.33)		0.85 (0.56–1.27)	
Dominant	AA	AC/CC	0.90 (0.71–1.14)	0.39	0.89 (0.70–1.14)	0.36
Recessive	AA/AC	CC	0.93 (0.63–1.37)	0.71	0.89 (0.60–1.31)	0.56
Overdominant	AA/CC	AC	0.93 (0.73–1.18)	0.53	0.93 (0.74–1.19)	0.58
Log-additive			0.93 (0.77–1.11)	0.41	0.91 (0.76–1.10)	0.33
**rs10505248**						
Codominant	A/A	A/G	1.20 (0.91–1.60)	0.36	0.95 (0.72–1.26)	0.94
		G/G	1.31 (0.64–2.70)		1.00 (0.47–2.11)	
Dominant	A/A	A/G-G/G	1.21 (0.93–1.59)	0.16	0.96 (0.73–1.25)	0.74
Recessive	A/A-A/G	G/G	1.21 (0.93–1.59)	0.55	1.01 (0.48–2.13)	0.98
Overdominant	A/A-G/G	A/G	1.19 (0.90–1.58)	0.22	0.95 (0.72–1.26)	0.73
Log-additive			1.18 (0.94–1.49)	0.16	0.97 (0.76–1.22)	0.78

SNP, single nucleotide polymorphism; CHD, coronary heart disease; IS, ischemic stroke. ^*^P < 0.008 (after adjusting for 6 independent tests by the Bonferroni correction).

The genotypes of the rs2954029 SNP were also associated with increased risk of IS in different genetic models: dominant model: TA/AA *vs*. AA (OR = 1.48, 95% CI = 1.14–1.90, *P* = 0.0026); and log-additive model: A *vs*. T (OR = 1.30, 95% CI = 1.09–1.55, *P* = 0.0039).

### Genotypes and serum lipid levels

**Figure 3:**
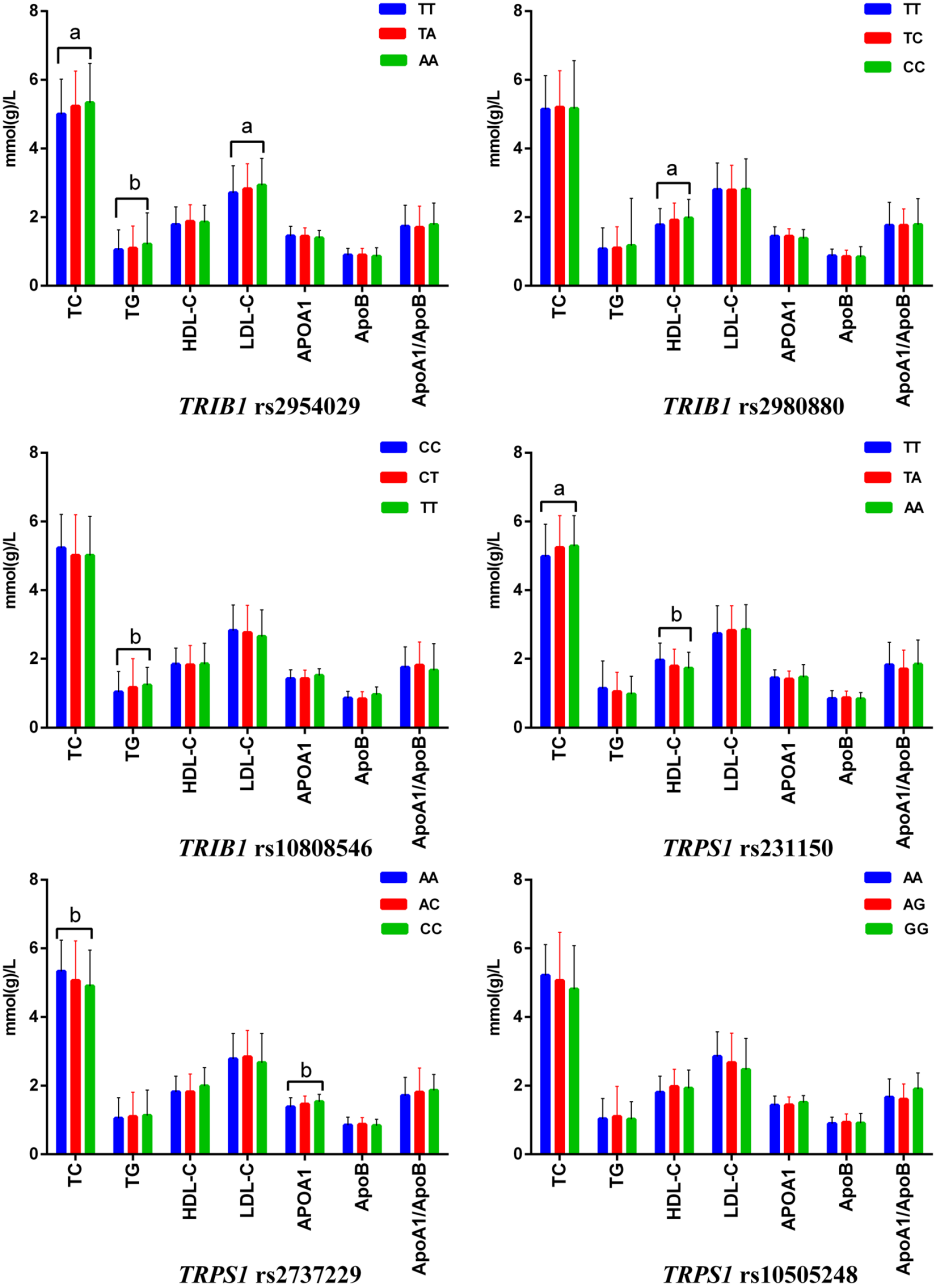
Genotypes of the six *TRIB1* and *TRPS1* SNPs and serum lipid levels in controls. TC, total cholesterol; TG, triglyceride; HDL-C, high-density lipoprotein cholesterol; LDL-C, low-density lipoprotein cholesterol; ApoA1, apolipoprotein A1; ApoB, apolipoprotein B; ApoA1/ApoB, the ratio of apolipoprotein A1 to apolipoprotein B. The value of triglyceride is presented as the median (interquartile range), and the difference among the genotypes was determined by the Kruskal-Wallis test. ^a^*P* < 0.008 (after adjusting for 6 independent tests by the Bonferroni correction) and ^b^*P* < 0.001.

The association of the six SNPs and serum lipid concentrations in controls is shown in Fig. 3. Several SNPs were associated with TC (rs2954029, rs231150 and rs2737229), TG (rs2954029 and rs10808546), low-density lipoprotein cholesterol (LDL-C, rs2954029), HDL-C (rs2980880 and rs231150) and ApoA1 (rs2737229). The minor allele carriers had higher levels of TC (rs2954029 and rs231150), TG (rs2954029 and rs10808546), LDL-C (rs2954029), HDL-C (rs2980880) and ApoA1 (rs2737229); and lower levels of TC (rs2737229), and HDL-C (rs231150) than the minor allele non-carriers.

### Haplotype and the risk of CHD and IS

**Figure 4:**
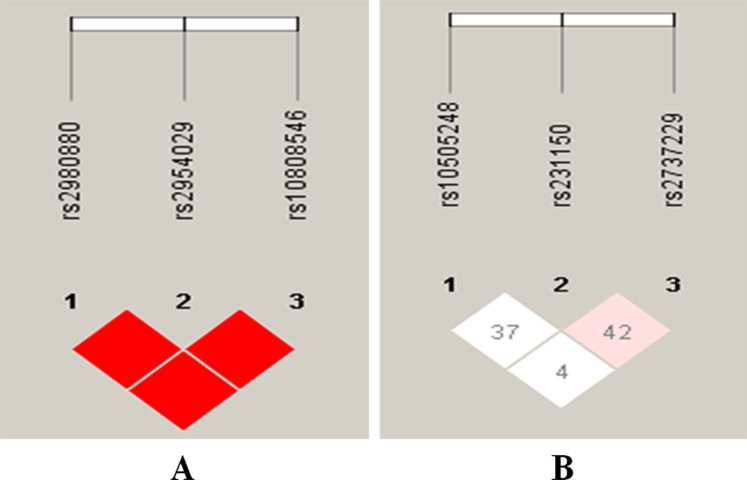
The linkage disequilibrium (LD) of the *TRIB1* and *TRPS1* SNPs.

There was strong linkage disequilibrium (LD) among the rs2954029, rs2980880 and rs10808546 SNPs in controls and patients (*D*′ = 1.0 for all, Fig. 4A) but weak LD among the rs231150, rs2737229 and rs10505248 SNPs (*D*′ < 0.5 for all, Fig. 4B). Thus, haplotype analyses among the rs2954029, rs2980880 and rs10808546 SNPs and the associations of their haplotypes and the risk of CHD and IS were performed. Four main haplotypes are shown in Table 4. The haplotype of A-C-C (in the order of rs2954029, rs2980880 and rs10808546 SNPs) was associated with increased risk for CHD (adjusted OR = 1.37, 95%CI = 1.11–1.70, *P* = 0.0036) and IS (adjusted OR = 1.31, 95%CI = 1.06–1.61, *P* = 0.01). The haplotype of A-T-C was associated with increased risk for CHD (adjusted OR = 1.63, 95% CI = 1.25–2.13, *P* < 0.001).

**Table 4:** Haplotype frequencies of the three *TRIB1* SNPs and the risk of CHD and IS.

Haplotype	Control Frequency	Frequency	CHD	*P*	Frequency	IS	*P*
			OR (95% CI)			OR (95% CI)	
T-T-C	0.4456	0.3895	1.00		0.4014	1.00	
A-C-C	0.2672	0.2816	1.37 (1.11–1.70)	0.0036	0.3083	1.31 (1.06–1.61)	0.01
T-T-T	0.1691	0.1186	1.16 (0.91–1.48)	0.22	0.1546	1.01 (0.79–1.29)	0.93
A-T-C	0.1176	0.1620	1.63 (1.25–2.13)	3E-4	0.1356	1.29 (0.97–1.70)	0.079

CHD, coronary heart disease; IS, ischemic stroke. The haplotypes consist of three alleles in the order of rs2954029, rs2980880 and rs10808546 SNPs.

### Gene-environment interactions on the risk of CHD and IS

**Figure 5:**
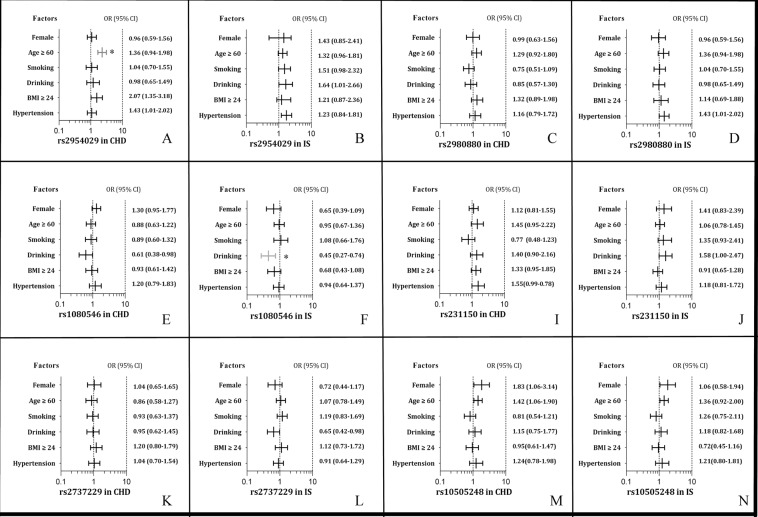
The interactions of the six SNPs and gender, age, drinking, smoking, BMI and hypertension on the risk of CHD and IS. CHD, coronary heart disease; IS, ischemic stroke. *P < 0.001.

The interactions of gene-environment on the risk of CHD and IS are shown in Fig. 5. The rs2954029TA/AA-age (>60 year) interaction increased the risk of CHD (OR = 2.12, 95% CI = 1.53–2.94, *P*_I_ = 0.0014), whereas the rs10808546CT/TT-drinking interaction decreased the risk of IS (OR = 0.45, 95% CI = 0.27–0.74, *P*_I_ = 0.00084).

### Gene-gene interactions on the risk of CHD and IS

**Figure 6:**
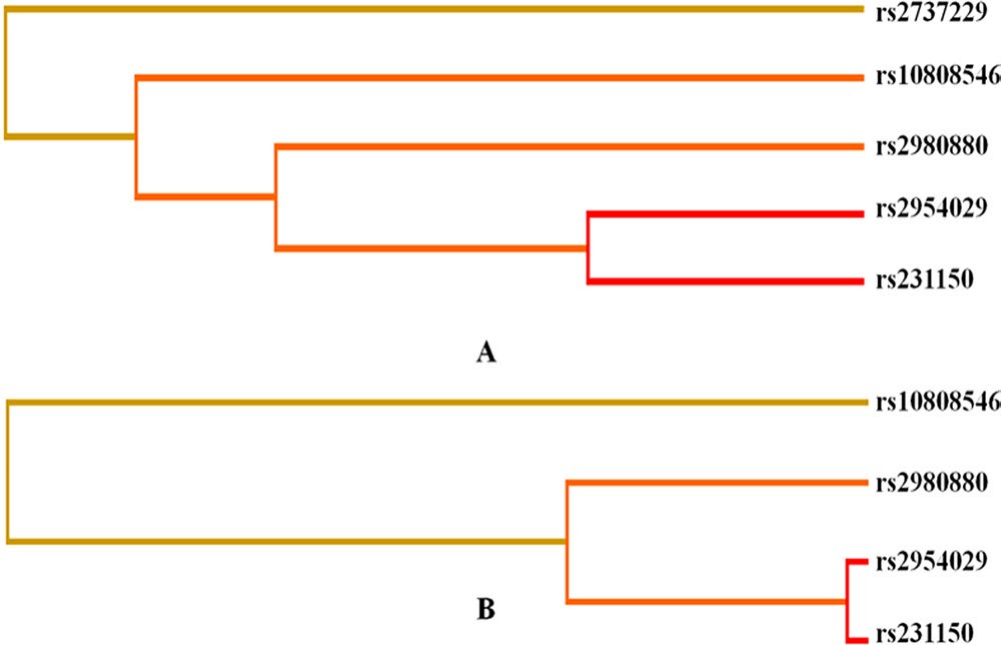
Diagram for gene-gene interactions on the risk of CHD (**A**) and IS (**B**). Red color, strong interaction; yellow color, weak interaction.

The interactions of gene-gene on the risk of CHD and IS are displayed in Fig. 6 and Tables 5 and 6. The interactions of the rs2954029-rs231150 on the risk of CHD and IS were relatively strong, whereas the interactions of the rs2954029-rs231150-rs10808546 and rs2954029-rs231150-rs10808546-rs2737229 on the risk of CHD, and the, rs2954029-rs231150-rs10808546 and rs2954029-rs231150-rs10808546-rs2980880 on the risk of IS were relatively weak (interaction strength: red color > yellow color).

**Table 5:** Gene-gene interactions on the risk of CHD. *P* value based on 1000 permutations; MDR, multifactor dimensionality reduction; CVC, Cross-validation consistency.

Model	Traing Bal.Acc	Testing Bal.Acc	CVC	*P*
rs231150	0.5371	0.4963	6/10	0.467
rs2954029-rs231150	0.5466	0.5432	10/10	0.037
rs2954029-rs231150-rs10808546	0.5680	0.5357	7/10	0.069
rs2954029-rs231150 -rs10808546-rs2737229	0.5851	0.5493	6/10	0.031

**Table 6:** Gene-gene interactions on the risk of IS.

Model	Traing Bal.Acc	Testing Bal.Acc	CVC	*P*
rs2954029	0.5377	0.5377	10/10	0.234
rs2954029-rs231150	0.6269	0.6155	7/10	0.003
rs2954029-rs231150-rs10808546	0.6518	0.6518	10/10	0.001
rs2954029-rs231150-rs10808546-rs2980880	0.6548	0.6473	9/10	0.001

*P* value based on 1000 permutations; MDR, multifactor dimensionality reduction; CVC, Cross-validation consistency.

## Discussion

In the present study, we showed that the genotype and allele frequencies of some *TRIB1* and *TRPS1* SNPs were different between controls and patients (CHD, rs2954029 and rs231150; IS, rs2954029 and rs2980880). Several SNPs were associated with TC (rs2954029, rs231150 and rs2737229), TG (rs2954029 and rs10808546), LDL-C (rs2954029), HDL-C (rs2980880 and rs231150) and ApoA1 (rs2737229) levels in controls. Genetic association analyses also showed that the SNPs of rs2954029 and rs231150 were associated with increased risk of CHD, and the SNP of rs2954029 was associated with increased risk of IS. The SNPs of rs2954029, rs2980880 and rs10808546 were strong LD in controls and patients. Four main haplotypes among three SNPs were detected. The haplotypes of rs2954029A-rs2980880C-rs10808546C and rs2954029A-rs2980880T-rs10808546C were associated with increased risk for CHD, whereas the haplotype of rs2954029A-rs2980880C-rs10808546C was associated with increased risk for IS. The rs2954029TA/AA-age (>60 year) interaction increased the risk of CHD, whereas the rs10808546CT/TT-drinking interaction decreased the risk of IS. The interactions of SNP-SNP in *TRIB1* and *TRPS1* on the risk of CHD and IS were also observed.

Previous studies have showed that several SNPs in *TRIB1* were associated with one or more lipid parameters^18,25–29^ and cardiovascular disease^13,18,25,30^. However, not all researches have consistent findings. A GWAS^18^ conducted in >100,000 individuals showed that the rs2954029A was associated with increased TG, TC and LDL-C levels, decreased HDL-C levels and increased risk of CHD. In another study, Varbo *et al*.^25^ proved that the rs2954029A allele was also associated with increased ApoB levels. However, a replicated study^26^ performed in the Indian population found that the rs2954029A allele carriers had higher HDL-C levels than the rs2954029A allele non-carriers. In addition, Kiran *et al*.^28^ demonstrated that the rs2980880C allele in *TRIB1* was associated with increased HDL-C levels in American. Previous studies^13,29^ also showed that the rs10808546T allele was associated with increased LDL-C and TG levels, lowed HDL-C levels and increased risk of CHD. In the present study, we found that the rs2954029A allele was associated with increased TC, TG and LDL-C levels and risk of CHD. What’s more, we also first identified that the rs2954029A allele carriers had higher risk of IS than the rs2954029A allele non-carriers, which has not been reported previously. In addition, the rs2980880C and rs1080880T alleles were associated with increased HDL-C and TG levels; respectively. These results were partly consistent with previous studies in other populations.

Studies showed that several SNPs in or near *TRPS1* were associated with TC, HDL-C or CHD. However, the association was not concordant in different populations. The minor C allele of rs2737229 SNP was associated with decreased TC levels in European and East Asian, but with increased TC levels in South Asian^18^. Another study^31^ showed that the rs10505428 SNP was associated with HDL-C levels. Lee *et al*.^23^ found that the minor rs231150A allele was associated with increased risk of CHD. In the present study, we showed that the rs2737229C allele was associated with low TC and high ApoA1 levels. The minor A allele of the rs231150 was associated with increased TC and decreased HDL-C levels and increased risk of CHD. We did not find that the rs1050824 SNP was associated with serum lipid profiles. The reasons for these diverse findings remain unclear, it may be owing to the impact of other uncertain variants and the other potential influence factors, such as differences in dietary habits. Another possible reason is that the sample size may not enough to detect the exact association. Therefore, further investigations with larger sample size are needed to confirm the association.

The increased risk of CHD and IS in *TRIB1* rs2954029TA/AA versus TT genotypes is possibly due to the combined increase of both TC and LDL-C. The cholesterol in both LDL-C and TC may accumulate in the arterial intima^32^, which may result in the atherosclerosis, the pathological basis of both CHD and IS^4^. However, the possible that the increased TG levels contributing to the development of atherosclerosis, CHD or IS cannot be excluded. Douvris *et al*.^33^ have demonstrated the interplay between *TRIB1*-associated locus (TRIBAL, a novel locus) and *TRIB1*. TRIBAL was identified as a risk locus for dyslipidemia in the genome wide association studies. It responded to altered expression of *TRIB1*, harbored a risk SNP (rs2001844) that was an eQTL for *TRIB1* expression, and associated with plasma TG concentrations. Another research^34^ demonstrated that the minor allele of rs6982502 SNP in this regulatory sequence was a risk allele for increasing plasma lipid levels and non-alcoholic fatty liver disease (NAFLD) reduced the activity of the *TRIB1* promoter. TRIB1 deficiency increases plasma cholesterol and TGs in mice and overexpression of *TRIB1* in mouse liver reduces these factors. In addition, studies *in vivo* mouse have showed that overexpression of *TRIB1* causes decrease of serum TC, TG, LDL, very-low-density lipoprotein (VLDL), and ApoB levels^35^ and that inactivation of *TRIB1* results in mixed hyperlipidemia by increasing hepatic lipogenesis and VLDL secretion^35^. *TRIB1* encodes tribble-1, a protein with a regulatory effect on mitogen-activated protein kinase^36^. It may be through this pathway that *TRIB1* influences lipid metabolism, resulting in dyslipidemia, CHD and IS. But the exact mechanism is still not unclear. It has also been suggested that *TRIB1* regulates chemotaxis and proliferation of smooth muscle cells in the arterial intima, and it may, through this, lead to CHD and IS independent of lipoproteins^37^.

The *TRPS1* encodes a transcription factor bound to a dynein light chain protein. The binding of the encoded protein affects subsequent binding to GATA consensus sequences, thereby suppressing its transcriptional activity. In this study, the *TRPS1* rs231150TA/AA genotype was associated with high risk of CHD. However, the mechanism for this association is unclear. It may be owing to the rs231150A allele was associated with increased TC and decreased HDL-C levels, both of which are the risk factors of CHD. *TRPS1* mRNA was down-regulated in obese adipocytes compared to non-obese adipocytes, suggesting that it might contribute to obesity pathology and lipid metabolism^38^. Furthermore, *TRPS1* has been showed to be involved in smooth muscle cell differentiation via transcriptional regulation^39^. Interestingly, in a transcriptome profiling study, *TRPS1* was found to be highly expressed in the macrophages of large atherosclerotic lesions in apoE-deficient mice^40^. All of these may be the possible mechanism for the *TRPS1* with CHD.

In the present study, we first explored the LD among the rs2954029, rs2980880 and rs10808546 SNPs and showed that there was high LD among them in controls and patients. Haplotype analyses among the three SNPs showed that the haplotype of rs2954029A-rs2980880C-rs10808546C was associated with increased risk of CHD and IS and the haplotype of rs2954029A-rs2980880T-rs10808546C was associated with increased risk for IS. However, these findings still need to be confirmed in the other populations with larger sample sizes.

The interactions of SNP-environment or SNP-SNP in *TRIB1* and *TRPS1* on the risk of CHD and IS have not been detected previously. In the present study, we revealed that when the rs2954029TA/AA genotype interacted with age (>60 year), it increased the risk of CHD, whereas the rs10808546CT/TT genotypes interacting with drinking decreased the risk of IS. The interactions of the rs2954029-rs231150 on the risk of CHD and IS were relatively strong, whereas the interactions of the rs2954029-rs231150-rs10808546 and rs2954029-rs231150-rs10808546-rs2737229 on the risk of CHD, and the rs2954029-rs231150-rs10808546 and rs2954029-rs231150-rs10808546-rs2980880 on the risk of IS were relatively weak. This is the first report to demonstrate an interaction of the *TRIB1* and *TRPS1* SNP-environment or SNP-SNP on the risk of CHD and IS in a Chinese Han population.

There are some potential limitations in our study. Firstly, the sample size is small compared with lots of previous GWASes. Secondly, some clinical characteristics were significantly different between the patients and controls. Although some confounders have been adjusted for the statistical analyses, we could not completely eliminate the potential influences of these factors on the results. Thirdly, the association of the rs2954029, rs2980880, rs1080880, rs2737229 and rs10505248 SNPs and serum lipid levels in CHD and IS patients was not analyzed because of the interference of lipid-lowering drugs. Finally, it is now generally accepted that both CHD and IS are the complex diseases caused by multiple environmental and genetic factors and their interactions. Although we have detected the association of the rs2954029, rs2980880, rs1080880, rs2737229 and rs10505248 SNPs in *TRIB1* and *TRPS1* and the risk of CHD and IS, other genetic variants are not detected and analyzed together, and this may result in some misinterpretation of our results.

In conclusion, the results of the present study showed that the genotype and allele frequencies of several SNPs were different between controls and patients (CHD, rs2954029 and rs231150; IS, rs2954029 and rs2980880). Several SNPs were associated with TC (rs2954029, rs231150 and rs2737229), TG (rs2954029 and rs10808546), LDL-C (rs2954029), HDL-C (rs2980880 and rs231150) and ApoA1 (rs2737229) levels in controls. Genetic association analyses also showed that the SNPs of rs2954029 and rs231150 were associated with increased risk of CHD (rs2954029 and rs231150) and IS (rs2954029). The haplotypes of rs2954029A-rs2980880C-rs10808546C and rs2954029A-rs2980880T-rs10808546C were associated with increased risk for CHD, whereas the haplotype of rs2954029A-rs2980880C-rs10808546C was associated with increased risk for IS. The rs2954029TA/AA-age (>60 year), rs10808546CT/TT-drinking and SNP-SNP (rs2954029-rs231150) interactions on the risk of CHD and IS were also observed.

## Materials and Methods

### Study patients

A total of 1146 unrelated patients with CHD (*n* = 593) and IS (*n* = 553) were recruited from hospitalized patients in the First Affiliated Hospital, Guangxi Medical University from Jun. 1, 2015 to Nov. 31, 2017. The diagnostic criteria for CHD were based on the 2013 ESC^41,42^ criteria: (1) typical ischemic chess pain; (2) typical discomfort accompanied by electrocardiographic change, including ST-segment depression or elevation of ≥0.5 mm, T-wave inversion of ≥3 mm in ≥3 leads or left bundle branch block; (3) cardiac markers, just as creatinine kinase-MB, troponin T and high-sensitivity C-reactive protein, were normal; (4) If justify by coronary angiography, patients were individual to significant coronary stenosis (≥50%) in at least either one of the three main coronary arteries or their major branches (branch diameter ≥2 mm). The coronary angiograms were reviewed by two independent angiographers. For a vessel to be scored, stenosis ≥50% had to be noted in an epicardial coronary vessel of interest or in one of its major branches. In the event of discordance of the number of vessels scored between the two reviewers, angiograms were scored by a third independent reviewer. (5) Angiographic severity of disease was classified according to the number of coronary vessels with significant stenosis (luminal narrowing ≥50%) as one-, two-, or three-vessel disease in the three major coronary arteries. Angiographers were blinded to the results of the genotypes. The diagnosis of IS met the criteria approved at the Fourth National Cerebrovascular Disease Conference in 1995. At least 2 clinically experienced physicians made the final diagnosis through the characteristics of clinical syndrome, brain computed tomography (CT), and magnetic resonance imaging (MRI). The patients with IS were excluded if IS was caused by transient ischemic attack, hemorrhagic cerebral infarction, cardiogenic cerebral embolism, tumors, cardiovascular malformations, peripheral arterial occlusive disease, trauma, drugs, blood or infectious diseases, or they had been taking lipid-lowering drugs within the last half of the year. According to the Trial of Org 10172 in Acute Stroke Treatment (TOAST) system^43^, the selected patients were divided into 2 subgroups: the LAA subgroup and the SAO subgroup. The recruited patients were three generations of Han people as in our previous^12^, which were confirmed by Y chromosome and mitochondrial diversity studies^44,45^. The selected IS patients who had a past history of CHD and the selected CHD patients who had a past history of IS were also excluded from the study.

### Control subjects

A total of 625 control subjects matched by age, gender, and ethnic group were randomly selected from the healthy adults who underwent periodical medical check-up at the Physical Examination Center of the First Affiliated Hospital, Guangxi Medical University during the same period when CHD and IS patients were recruited. The controls were free of CHD and IS by questionnaires, history-taking, and clinical examination. The examination comprised physical examination, blood sampling, electrocardiography, chest X-ray, and Doppler echocardiography. All enrolled individuals were Han Chinese from Guangxi, the People’s Republic of China. Information on demography, socioeconomic status, medical history, and lifestyle factors was collected by trained research staff with standardized questionnaires for all participants. This study was approved by the Ethics Committee of, the First Affiliated Hospital, Guangxi Medical University, and written informed consent was obtained from each participant before data collection. The reported investigations were in accordance with the principles of the Declaration of Helsinki.

### Biochemical measurements

Venous blood samples were collected from all subjects after at least 12 h of fasting. The levels of serum TC, TG, HDL-C, and LDL-C in samples were determined by enzymatic methods with commercially available kits, Tcho-1, TG-LH (RANDOX Laboratories Ltd., Ardmore, Diamond Road, Crumlin Co., Antrim, UK, BT29 4QY), Cholestest N HDL, and Cholestest LDL (Daiichi Pure Chemicals Co., Ltd., Tokyo, Japan), respectively. Serum apolipoprotein (Apo) A1 and ApoB levels were detected by the immunoturbidimetric immunoassay (RANDOX Laboratories Ltd.). All determinations were performed with an autoanalyzer (Type 7170 A; Hitachi Ltd., Tokyo, Japan) in the Clinical Science Experiment Center of the First Affiliated Hospital, Guangxi Medical University^46,47^.

### Diagnostic criteria

The normal values of serum TC, TG, HDL-C, LDL-C, ApoA1, and ApoB levels, and the ratio of ApoA1 to ApoB in our Clinical Science Experiment Center were 3.10–5.17, 0.56–1.70, 0.91–1.81, 2.70–3.20 mmol/L, 1.00–1.78, 0.63–1.14 g/L, and 1.00–2.50, respectively^48,49^. The individuals with TC > 5.17 mmol/L, and/or TG > 1.70 mmol/L were defined as hyperlipidemic^50,51^. Hypertension was defined according to the criteria outlined by the 1999 World Health Organization—International Society of Hypertension Guidelines for the management of hypertension^52^. Uncontrolled hypertension was defined as a systolic blood pressure of 140 mmHg or higher and/or a diastolic blood pressure of 90 mmHg or higher. The subjects with systolic blood pressure of only 140 mmHg or higher but a diastolic blood pressure of <90 mmHg were diagnosed as isolated systolic hypertension. Normal weight, overweight, and obesity were defined as a BMI < 24, 24–28, and >28 kg/m^2^, respectively^53^.

### SNP selection

The six SNPs in *TRIB1* and *TRPS1* were selected on the basis of the following criteria: (1) Tagging SNPs were established by Haploview (Broad Instituteof MIT and Harvard, Cambridge, MA, USA, version 4.2); (2) SNPs information was obtained from NCBI dbSNP Build 132 (http://www.ncbi.nlm.nih.gov/SNP/); (3) SNPs were restricted to minor allele frequency (MAF) > 5%; and (4) SNPs might be associated with the plasma lipid levels or cardiovascular disease in recent studies^13,18,23,24,26,28,54–56^. (5) the target SNP region should be adequately replicated by PCR, and the polymorphic site should have commercially available restriction endonuclease enzyme to be genotyped with RFLP.

### Genotyping

Genomic DNA of the samples was isolated from peripheral blood leukocytes according to the phenol-chloroform method^57,58^. Genotyping of six mutations was performed by PCR-RFLP and determined by Sanger sequencing. The characteristics of each SNP and the details of each primer pair, annealing temperature, length of the PCR products and restriction fragment are summarized in Supplemental Tables 1, 2 and Supplemental Figs 1, 2. The PCR products of the samples (two samples of each genotype) were sequenced with an ABI Prism 3100 (Applied Biosystems, International Equipment Trading Ltd., Vernon Hill, IL, USA) in Shanghai Sangon Biological Engineering Technology & Services Co., Ltd., China.

### Statistical analyses

We employed the statistical software package SPSS 22.0 (SPSS Inc., Chicago, IL, USA) to analyze the data. Quantitative variables were expressed as the means ± standard deviation (TG levels were presented as medians and interquartile ranges and were analyzed by Wilcoxon- Mann-Whitney test because they were not a normal distribution). Qualitative variables are presented as percentages. Allele frequency was determined via direct counting, and the standard goodness-of-fit test was used to test HWE. The difference in genotype distribution and sex ratio between the groups was used a chi-square analysis. The general characteristics between patient and control groups were tested by the Student’s unpaired *t*-test. The association of genotypes and serum lipid parameters was tested by analysis of covariance (ANCOVA). Any variants associated with the serum lipid parameter at a value of *P* < 0.008 (corresponding to *P* < 0.05 after adjusting for six independent tests by the Bonferroni correction) were considered statistically significant. After adjusting for the age, gender, BMI, smoking, and alcohol consumption, we employed unconditional logistic regression to evaluate the correlation between the risk of CHD and IS and genotypes. The same methods were used to calculate the odds ratio (OR) and 95% confidence interval (95% CI). The interactions of six SNPs with environment exposures, including sex, age, alcohol consumption, cigarette smoking and BMI ≥ 24 kg/m^2^ on the risk of CHD and IS were employed ANOVA two-way factorial design and unconditional logistic regression after controlling for potential confounders. A *P*_I_ ≤ 0.0017 was considered statistically significant after Bonferroni correction (corresponding to *P* < 0.05 after adjusting for five environment exposures multiplied by six independent tests by the Bonferroni correction). Haploview (Broad Institute of MIT and Harvard, USA, version 4.2) analyzed the haplotype frequencies and pairwise LD among the detected SNPs.

## Supplementary information

Dataset 1

Dataset 2
