# Supplementary material for: TRIB1 and TRPS1 variants, G × G and G × E interactions on serum lipid levels, the risk of coronary heart disease and ischemic stroke
Source: Sci Rep. 2019 Feb 20;9:2376. doi: 10.1038/s41598-019-38765-7 (PMC6382757; doi:10.1038/s41598-019-38765-7)
Supplement: Supplementary file 1 — Dataset 1 [file 41598_2019_38765_MOESM1_ESM.docx]

***TRIB1* and *TRPS1* variants, G×G and G×E interactions on serum lipid levels, the risk of coronary heart disease and ischemic stroke**

Qing-Hui Zhang^1^, Rui-Xing Yin^1^, Wu-Xian Chen^1^, Xiao-Li Cao^2^ & Jin-Zhen Wu^1^

^1^Department of Cardiology, Institute of Cardiovascular Diseases, The First Affiliated Hospital, Guangxi Medical University, Nanning 530021, Guangxi, People’s Republic of China. ^2^ Department of Neurology, The First Affiliated Hospital, Guangxi Medical University, Nanning 530021, Guangxi, People’s Republic of China.


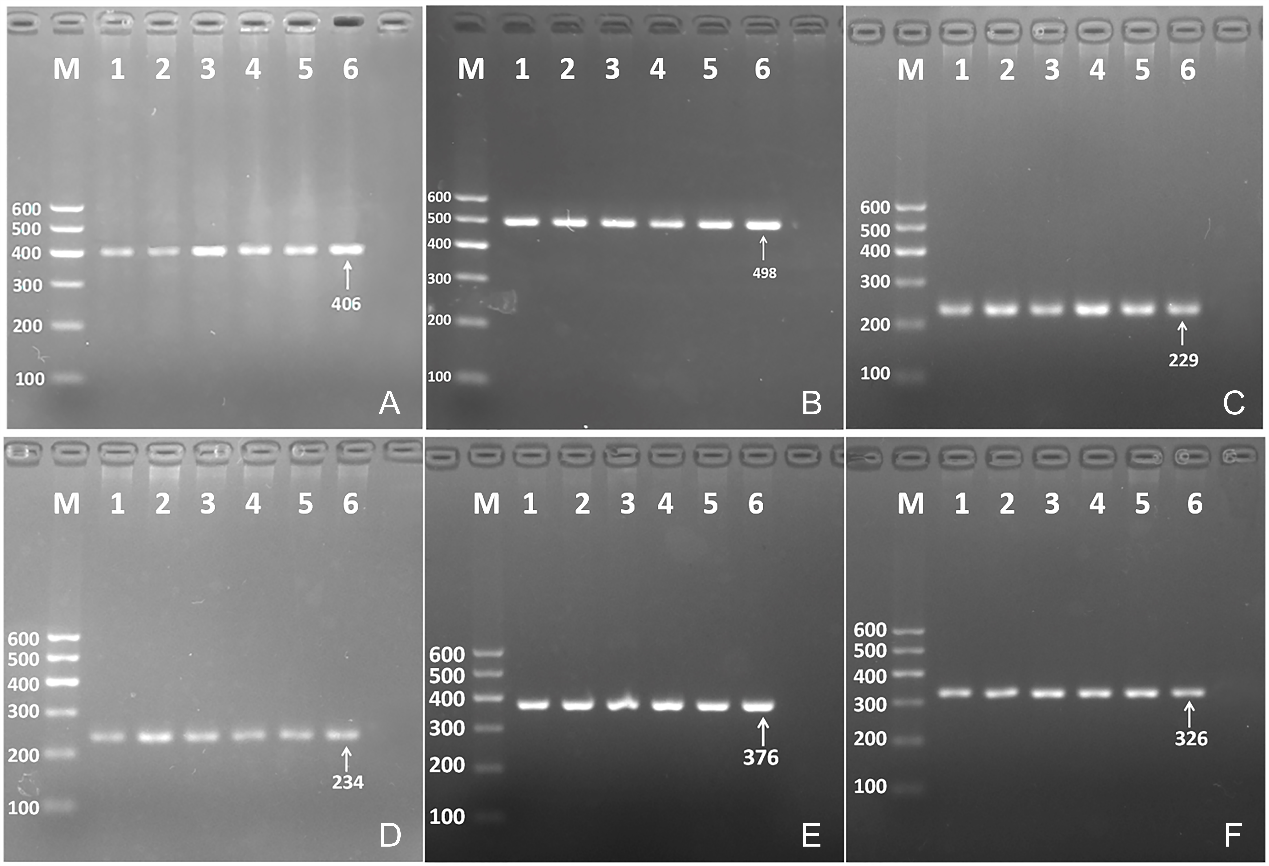


**Supplemental Figure 1. Agarose gel electrophoresis (2%) of PCR products of the *TRIB1* and *TRPS1* SNPs.** Lane M: DNA ladder 100bp; PCR amplicon of (A)*TRIB1* rs2954029, (B) *TRIB1* rs2980880, (C) *TRIB1* rs10808546, (D) *TRPS1* rs231150, (E) *TRPS1* rs2737229 and (F) *TRPS1* rs10505248 SNPs were 406-, 498-, 229-, 234-, 376- and 326-bp nucleotide sequences; respectively.


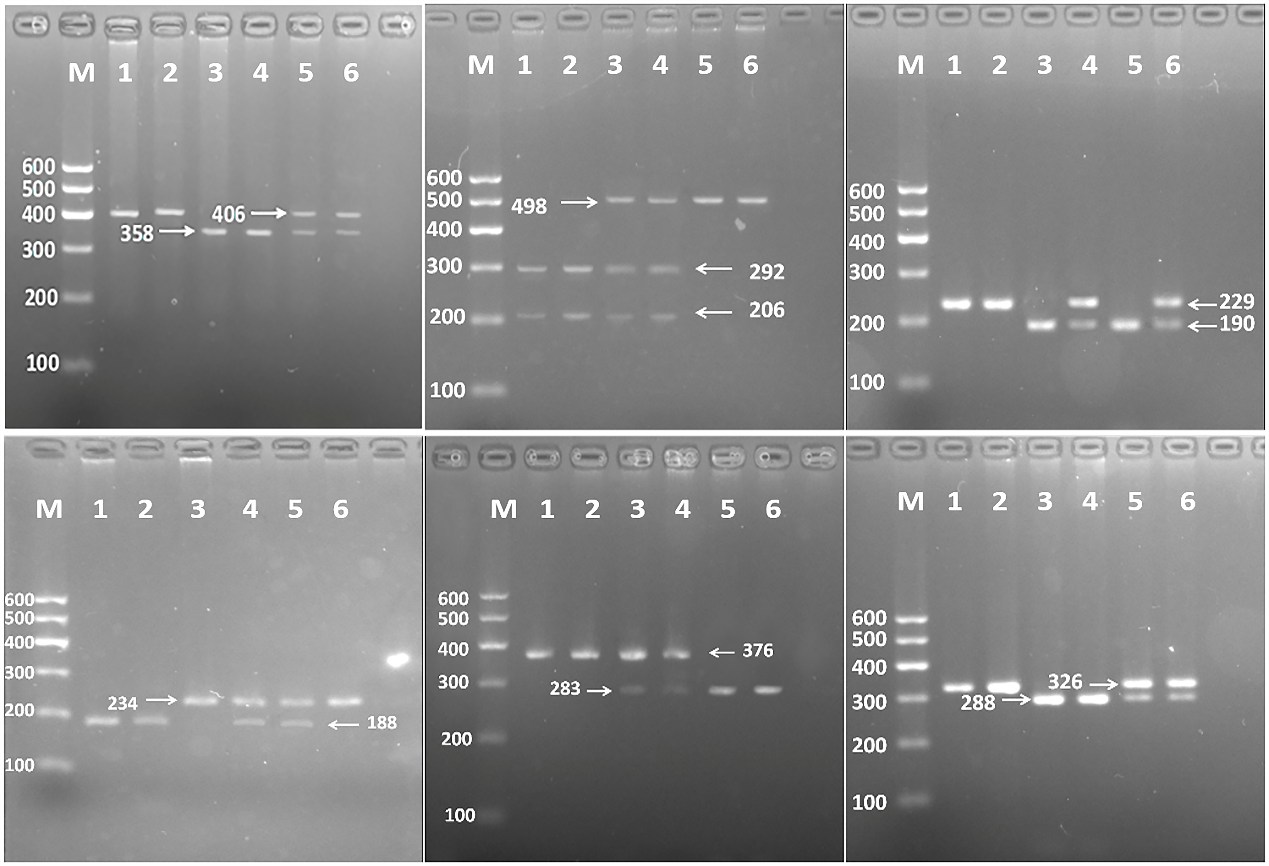


**Supplemental Figure 2. Agarose gel electrophoresis (2%) of genotyping of the *TRIB1*and *TRPS1* SNPs**. Lane M: DNA ladder100bp. The genotypes of 6 SNPs were as follow: (A) *TRIB1* rs2954029: AA (Lanes 1 and 2, 406-bp); TT (lanes 3 and 4, 358- and 48-bp); and AT genotype (lanes 5 and 6, 406-,358- and 48-bp). (B) *TRIB1* rs2980880: CC (lanes 1 and 2, 292- and 206-bp); CT (lanes 3 and 4, 498-, 292- and 206-bp); and TT genotype (lanes 5 and 6, 498-bp). (C) *TRIB1* rs10808546: CC (lanes 1 and 2, 229-bp); TT (lanes 3 and 5, 190- and 39-bp); and CT genotype (lanes 4 and 6, 229-, 190- and 39-bp). (D) *TRPS1* rs231150: TT (lanes 1 and 2, 188- and 46-bp); AA (lanes 3 and 6, 243-bp); and TA genotype (lanes 4 and 5, 243-,188- and 46-bp). (E) *TRPS1* rs2737229: AA (lanes 1 and 2, 376-bp); GC (lanes 3 and 4, 376-, 283- and 113-bp); and GG (lanes 5 and 6, 283- and 113-bp). (F) *TRPS1* rs10505248: AA (lanes 1 and 2, 326-bp); GG (lanes 3 and 4 288- and 38-bp); and AG genotype (lanes 5 and 6, 326-, 288- and 38-bp). The less than 100-bp fragment was invisible in the gel owing to its fast migration speed.
