# Supplementary material for: TRIB1 and TRPS1 variants, G × G and G × E interactions on serum lipid levels, the risk of coronary heart disease and ischemic stroke
Source: Sci Rep. 2019 Feb 20;9:2376. doi: 10.1038/s41598-019-38765-7 (PMC6382757; doi:10.1038/s41598-019-38765-7)
Supplement: Supplementary file 2 — Dataset 2 [file 41598_2019_38765_MOESM2_ESM.docx]

***TRIB1* and *TRPS1* variants, G×G and G×E interactions on serum lipid levels, the risk of coronary heart disease and ischemic stroke**

Qing-Hui Zhang^1^, Rui-Xing Yin^1^, Wu-Xian Chen^1^, Xiao-Li Cao^2^ & Jin-Zhen Wu^1^

^1^Department of Cardiology, Institute of Cardiovascular Diseases, The First Affiliated Hospital, Guangxi Medical University, Nanning 530021, Guangxi, People’s Republic of China. ^2^ Department of Neurology, The First Affiliated Hospital, Guangxi Medical University, Nanning 530021, Guangxi, People’s Republic of China.

**Supplemental Table 1.** Characteristics of the *TRIB1* and *TRPS1* SNPs.

| **SNP ID (rs#)** | **Chr:Position** | **Contig** | **Contig Pos** | **SNP to Chr** | **MAF/Minor** |
| --- | --- | --- | --- | --- | --- |
| ***TRIB1*** |  |  |  |  |  |
| rs2954029 | 8:126490972 | NT_008046.16 | 39764521 | Fwd | T=0.4050/2028(1000 Genomes) |
| rs2980880 | 8:126480972 | NT_008046.16 | 39754521 | Rev | G=0.2891/1448(1000 Genomes) |
| rs10808546 | 8:126495818 | NT_008046.16 | 39769367 | Fwd | T=0.2861/1433(1000 Genomes) |
| ***TRPS1*** |  |  |  |  |  |
| rs231150 | 8:116420327 | NT_008046.16 | 29693876 | Fwd | A=0.4233/2120(1000 Genomes) |
| rs2737229 | 8:116648565 | NT_008046.16 | 29922114 | Fwd | A=0.4890/2449(1000 Genomes) |
| rs10505248 | 8: 116156852 | NT_008046.16 | 29430401 | Fwd | G=0.1627/815 (1000 Genomes) |

**Supplemental Table 2.** The sequences of forward and backward primers of the *TRIB1* and *TRPS1* SNPs.

| **SNP** | **Primer sequence** | **Annealing temperature** | **PCR product(bp)** | **Restriction enzyme/site** | **Restriction fragment (bp)** | **Allele** |
| --- | --- | --- | --- | --- | --- | --- |
| *TRIB1* |  |  |  |  |  |  |
| rs2954029 | ACCAAATATCCGTGCCATTTAC | 58°C | 406 | BstX2I | 406 | A |
|  | GAAATGACAATTTCAGCCAACA |  |  | R^GATCY | 358+48 | T |
| rs2980880 | AGGGTAGCAGTAAGGAAGAGC | 58°C | 498 | SmoI | 498 | T |
|  | GCTTCTCCATTCACCTGCTG |  |  | C^TYRAG | 292+206 | C |
| rs10808546 | GCCAAAATAGTCCCACTGCA | 59°C | 229 | [FaeI](http://rebase.neb.com/rebase/enz/FaeI.html) | 229 | C |
|  | GGCCTAAGATCTTCTATGAGCCT |  |  | CATG^ | 190+39 | T |
| ***TRPS1*** |  |  |  |  |  |  |
| rs231150 | TCTGGATCTGTTCTGCCTCA | 60°C | 234 | [BspMI](http://rebase.neb.com/rebase/enz/BspMI.html) | 243 | A |
|  | TGAGTGTCGAGTATTGGGTAATG |  |  | ACCTGCN4^ | 188+46 | T |
| rs2737229 | GTTCCTGTTTACTGCGCCAC | 60°C | 376 | [BaeI](http://rebase.neb.com/rebase/enz/BaeI.html) | 376 | A |
|  | CGCCCAGCCAAGTACTTAAC |  |  | ACN4GTAYCN10^ | 283+113 | C |
| rs10505248 | TGTTTCCTCCTGTGTCCTCA | 60°C | 326 | [RsaI](http://rebase.neb.com/rebase/enz/RsaI.html) | 326 | A |
|  | AAGAGGGAAAGGAAGAGGGG |  |  | GT^AC | 288+38 | G |
